# Discovery of novel virus sequences in an isolated and threatened bat species, the New Zealand lesser short-tailed bat (*Mystacina tuberculata*)

**DOI:** 10.1099/vir.0.000158

**Published:** 2015-08

**Authors:** Jing Wang, Nicole E. Moore, Zak L. Murray, Kate McInnes, Daniel J. White, Daniel M. Tompkins, Richard J. Hall

**Affiliations:** ^1^​ Institute of Environmental Science & Research (ESR), at the National Centre for Biosecurity & Infectious Disease, PO Box 40158, Upper Hutt 5140, New Zealand; ^2^​ Department of Conservation, , 18–32 Manners Street, PO Box 6011, Wellington, New Zealand; ^3^​ Landcare Research, Private Bag 1930, Dunedin, New Zealand

## Abstract

Bats harbour a diverse array of viruses, including significant human pathogens. Extensive metagenomic studies of material from bats, in particular guano, have revealed a large number of novel or divergent viral taxa that were previously unknown. New Zealand has only two extant indigenous terrestrial mammals, which are both bats, *Mystacina tuberculata* (the lesser short-tailed bat) and *Chalinolobus tuberculatus* (the long-tailed bat). Until the human introduction of exotic mammals, these species had been isolated from all other terrestrial mammals for over 1 million years (potentially over 16 million years for *M. tuberculata*). Four bat guano samples were collected from *M. tuberculata* roosts on the isolated offshore island of Whenua hou (Codfish Island) in New Zealand. Metagenomic analysis revealed that this species still hosts a plethora of divergent viruses. Whilst the majority of viruses detected were likely to be of dietary origin, some putative vertebrate virus sequences were identified. Papillomavirus, polyomavirus, calicivirus and hepevirus were found in the metagenomic data and subsequently confirmed using independent PCR assays and sequencing. The new hepevirus and calicivirus sequences may represent new genera within these viral families. Our findings may provide an insight into the origins of viral families, given their detection in an isolated host species.

## Introduction

Bats (order Chiroptera) are the second most diverse group of mammals with over 1200 species, accounting for more than 20 % of mammals (Simmons, 2005). They occur throughout most of the world except for the two polar regions. In the last decade, it has become increasingly apparent that bats are important natural reservoirs for emerging and re-emerging zoonotic viruses, due at least in part to roosting habitats, the formation of large colonies, adaptive immune systems, a long life span, and long-distance flying capability (Calisher *et al.*, 2006; Luis *et al.*, 2013). Bat-associated viruses are known to cause severe disease in humans, and rabies has been recognized for centuries in this regard. Many zoonotic viruses that have emerged recently are thought to have their origins in bats. For example, Chinese horseshoe bats (genus *Rhinolophus*) are thought to be the source of severe acute respiratory syndrome coronavirus, which emerged in South China via an intermediate host (caged civets) and then caused significant mortality in humans (Ge *et al.*, 2013; Lau *et al.*, 2005; Li *et al.*, 2005; Wang *et al.*, 2006). Evidence similarly points to the Egyptian tomb bat (*Taphozous perforatus*) as a potential source of Middle East respiratory syndrome coronavirus (Chan *et al.*, 2013b; Memish *et al.*, 2013; Milne-Price *et al.*, 2014). Other examples of pathogenic human viruses that may have emerged from bats (O'Shea *et al.*, 2014) include Ebola virus (Leroy *et al.*, 2005), Nipah virus (Chua *et al.*, 2000), Hendra virus (Murray *et al.*, 1995; Selvey *et al.*, 1995) and Australian bat lyssavirus (Warrilow, 2005). Bats are often asymptomatic during infection, but the disease can be severe for a new host species when cross-species transmission occurs (Chan *et al.*, 2013a; Smith & Wang, 2013). Surveys of the bat virome and monitoring of virus dynamics in bat populations can inform our fundamental understanding on how viral pathogens emerge and evolve, enabling strategies for the prevention of future outbreaks or pandemics.

The global diversity of viruses found in bats is still largely unknown, and it is thought that a great number of virus taxa are yet to be discovered (Anthony *et al.*, 2013). Viruses in native New Zealand bats are poorly studied, perhaps in part due to the absence of any significant association with disease, either in the bats themselves or in other species. Three bat species represent the entire indigenous terrestrial mammalian fauna of New Zealand, the long-tailed bat (*Chalinolobus tuberculatus*), the lesser short-tailed bat (*Mystacina tuberculata*), and the extinct greater short-tailed bat (*Mystacina robusta*) (King, 2005). *M. tuberculata* is thought to have lived in isolation for over 16 million years until the arrival of *C. tuberculatus* over 1 million years before the present (BP) (O'Donnell, 2001). Other terrestrial mammals have been introduced within the last 800 years by Polynesian explorers and Europeans (King, 2005). Given the relative isolation of these bats, it may be possible to make inferences on the origins of virus groups discovered within this species. For example, we have previously reported the discovery of an alphacoronavirus in *M. tuberculata* guano from a remote offshore island of New Zealand (Hall *et al.*, 2014a). Alphacoronaviruses exclusively infect mammals, so this virus either has been circulating in native bats for more than 1 million years or has arrived via other mammals that were introduced later by humans. The former hypothesis would be congruent with a recent study contending that coronaviruses have an ancient origin (Wertheim *et al.*, 2013), whereas previous molecular clocks estimate an origin for all coronaviruses of only 10 000 years BP (Woo *et al.*, 2012).

This study provides an analysis of metagenomic data in *M. tuberculata* from two bat guano samples collected from Codfish Island (Whenua hou) in New Zealand, revealing the presence of a large number of insect, plant and vertebrate viruses. Our aim was to determine whether this bat species was a host to potential bat pathogens, or zoonotic pathogens, that are relevant to human and wildlife health.

## Results

**Fig. 1.**
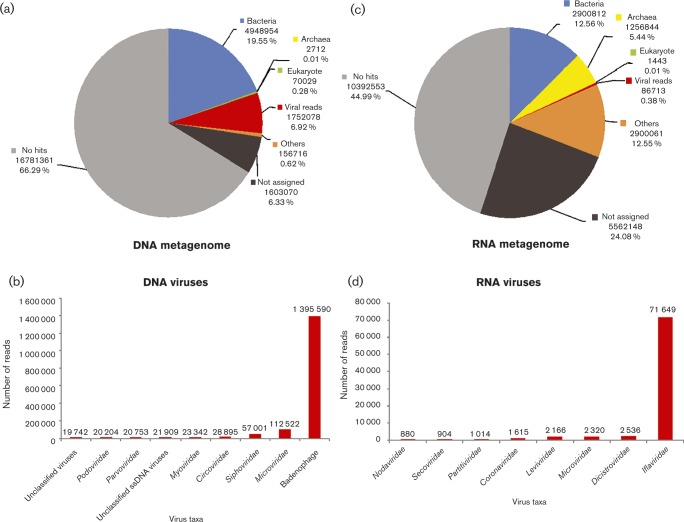
Metagenomic data summary from RAPSearch2 results. Overall summary of the taxonomic assignment for individual sequence reads from the metagenomic data of two New Zealand short-tailed bat guano samples for the combined DNA metagenome (a), DNA viruses (b), combined RNA metagenome (c) and RNA viruses (d).

For the two guano samples analysed by metagenomics, 25 314 920 and 23 100 574 sequence reads were generated in total for the DNA and RNA metagenomes, respectively. In the DNA metagenome, 19.55, 0.28 and 6.92 % of reads were assigned to bacteria, eukaryotes and viruses, respectively (Fig. 1a), with 79.65 % of virus reads assigned to bacteriophages (Fig. 1b). In the RNA metagenome, 44.99, 5.44 and 0.38 % of reads were assigned to bacteria, eukaryotes and viruses, respectively (Fig. 1c), with 82.63 % of virus reads assigned to the family *Flaviviridae* (Fig. 1d), a virus group known to infect insects and thus consistent with the insectivorous diet of *M. tuberculata*. Less than 2 % of all virus sequence reads showed similarity to vertebrate viruses, with significant RAPSearch2 matches to conserved gene regions used for taxonomic assignment (i.e. capsid and/or helicase genes) of papillomavirus, picornavirus, polyomavirus, calicivirus and hepevirus.

### Papillomaviridae

**Fig. 2.**
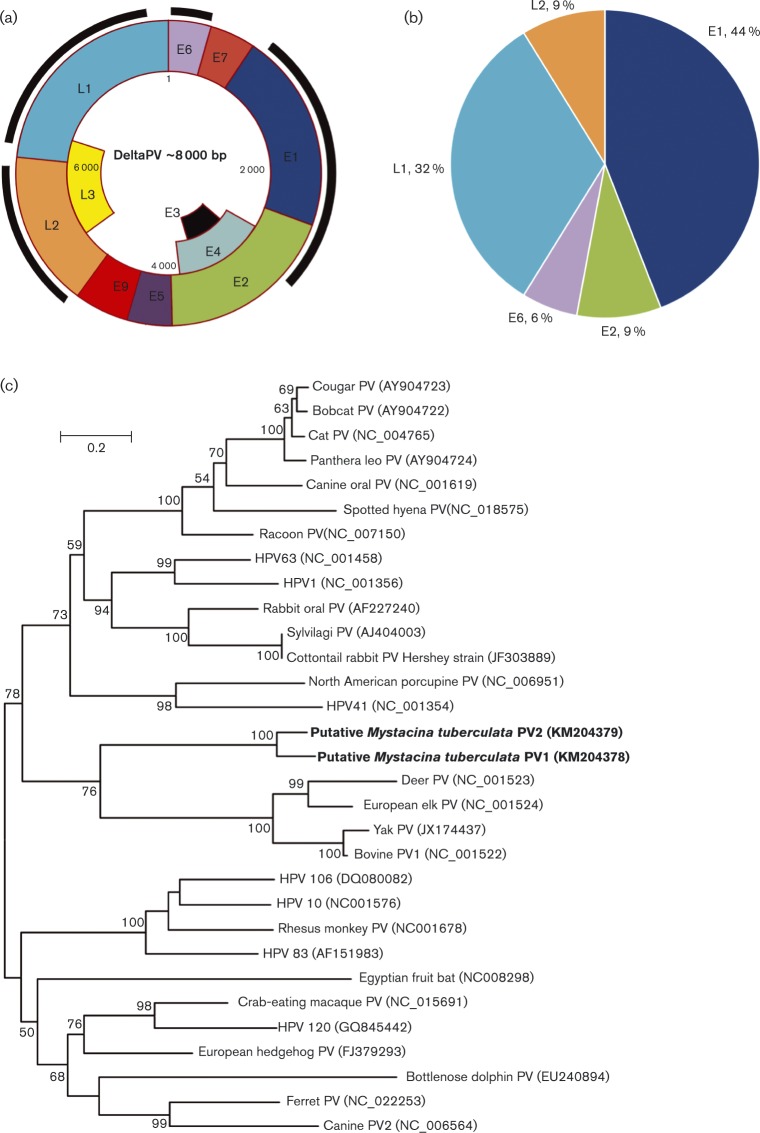
Identification of putative papillomavirus sequences. (a) Schematic representation of the location of contigs matching the papillomavirus genome, shown as the outer dark black line. Genome positions (bp) are shown inside. (b) Proportions of gene segments in the metagenomic data matching papillomavirus sequences. (c) Phylogenetic tree showing the relationship of the New Zealand bat papillomavirus L1 directly from the metagenomic data with that of other papillomaviruses. The New Zealand bat papillomavirus sequences were 172 aa (GenBank accession no. KM204379) and 173 aa (GenBank accession no. KM204378) respectively. The tree was compiled using the maximum-likelihood method and Lee and Gascuel model with 1000 bootstrap replicates. Bootstrap values < 50 % are not shown. Bar, number of amino acid substitutions per site. Asterisks (^*^) denote unclassified caliciviruses which have been suggested as new genera.

Papillomaviruses are non-enveloped, small dsDNA viruses that are capable of infecting all amniotes. In total, 105 sequence reads were identified as having similarity to papillomaviruses within the DNA metagenome, which assembled into 39 contigs matching E1, E2, E6, L1 and L2 genes (Fig. 2a, b). The sequences of the two L1 contigs, 518 and 523 bp, were confirmed by PCR and Sanger sequencing (GenBank accession nos. KM204378 and KM204379), and each was found in a different single guano sample. These sequences were then used for phylogenetic analysis. Based on these data, the two novel papillomavirus sequences (here named PV1 and PV2) grouped with deltapapillomaviruses (Fig. 2c). The L1 gene of PV1 (KM204378) shared 54.2 % amino acid sequence identity to its closest relative, bovine papillomavirus (GenBank accession no. NC001522). The L1 gene of PV2 (KM204379) showed 54.3 % similarity to its closest relative, deer papillomavirus (GenBank accession no. NC001523). The two New Zealand bat papillomaviruses had 86.1 % amino acid sequence identity for the partial L1 gene sequence.

### Polyomaviridae

**Fig. 3.**
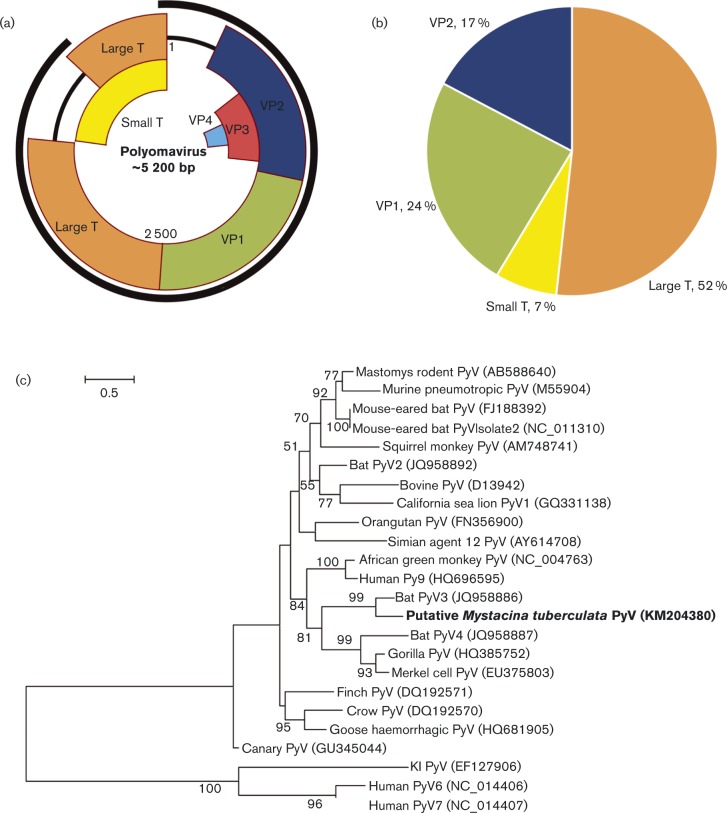
Identification of a putative PyV sequence. (a) Schematic representation of the location of contigs matching the PyV genome, shown as the outer dark black line. Genome positions (bp) are shown inside. (b) Proportions of gene segments in the metagenomic data matching PyV. (c) Phylogenetic tree showing the relationship of the New Zealand bat polyomavirus partial VP1 translated sequence of 203 aa (GenBank accession no. KM204380) directly from the metagenomic data with that of other polyomaviruses. The tree was compiled using the maximum-likelihood method and the Lee and Gascuel model with 1000 bootstrap replicates. Bootstrap values < 50 % are not shown. Bar, number of amino acid substitutions per site.

Polyomaviruses (PyVs) are small, circular dsDNA viruses. Their natural hosts are mammals and birds. In total, 493 sequence reads were identified in the DNA metagenome as having similarity to PyVs, which assembled into 29 contigs covering VP1, VP2, VP3, and the small T antigen and large T antigen genes (Fig. 3a, b). The presence of one of the VP1 contigs, 609 bp (GenBank accession no. KM204380), was confirmed in two out of the four samples using PCR and Sanger sequencing for a 187 bp subfragment of the contig. Phylogenetic analysis (Fig. 3c) showed that this novel bat PyV sequence grouped with a PyV found previously in South American bats (Fagrouch *et al.*, 2012). The VP1 gene (GenBank accession no. KM204380) shared 76.8 % identity at the amino acid sequence level with the closest relative, bat polyomavirus 4 (GenBank accession no. JQ958887). By mapping these putative PyV sequences to the reference genome of GenBank accession no. JQ958886, the consensus contig was shown to cover 92 % of the whole genome and included some minor gaps (Fig. 3a).

### Caliciviridae

**Fig. 4.**
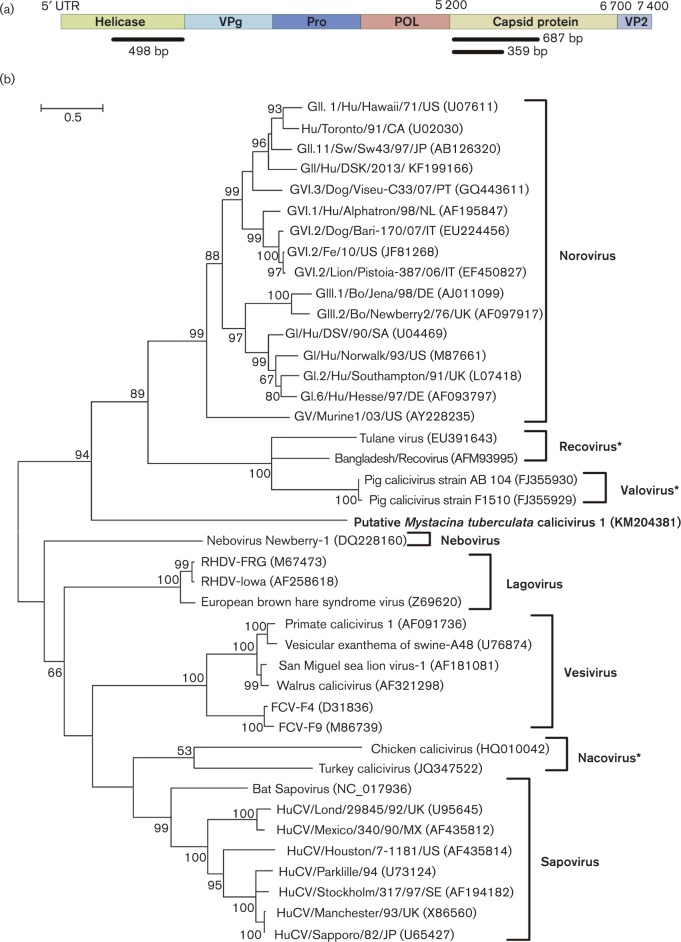
Identification of putative calicivirus sequences. (a) Schematic representation of the location of contigs matching the calicivirus genome, shown as the lower black bars. (b) Phylogenetic tree showing the relationship of the New Zealand bat calicivirus translated capsid sequence (GenBank accession no. KM204381) directly from the metagenomic data with that of other caliciviruses. The tree was compiled using the maximum-likelihood method and the Lee and Gascuel model with 1000 bootstrap replicates, including the New Zealand bat calicivirus partial capsid sequence of 228 aa (GenBank accession no. KM204381). Bootstrap values < 50 % are not shown. Bar, number of amino acid substitutions per site. Asterisks (*) denote unclassified caliciviruses which have been suggested as new genera.

A total of 68 sequence reads were identified in the RNA metagenome as having similarity to caliciviruses, which were then assembled into three contigs, two matching the capsid protein and one matching the helicase protein (Fig. 4a). Two capsid protein contigs of 687 and 359 bp in length (GenBank accession nos KM204381 and KM204382, respectively) and a contig containing a helicase gene with a length of 498 bp (GenBank accession no. KM204383) were identified. The presence of the larger contig (KM204381) could only be confirmed in one of the four guano samples, achieved using Sanger sequencing and PCR for a 302 bp subfragment of the metagenomic contig. Phylogenetic analysis of the larger capsid contig suggested that this virus sequence may be from a divergent calicivirus (Fig. 4b). This contig shared 30 % amino acid sequence identity with the closest calicivirus relative norovirus, GII.1/Hu/Hawaii/71/US (GenBank accession no. U07611).

Phylogenetic analysis of the helicase protein showed it to be so distinct from any other calicivirus that it always formed an outgroup, even with the inclusion of a distantly related picornavirus helicase (data not shown). It is clear that a viral helicase protein (or prophage remnant) is encoded in this sequence, as it belongs to the helicase superfamily 3 (SF-3). The protein appears to contain a Walker A (AIILT**G**PPGC**GKT**T) domain (shown in bold type), Walker B domain (IVVW**DD**) and motif 3 (FIIICS**N**F) that are characteristic of viral helicases (Hickman & Dyda, 2005). A subfragment of the metagenomic contig was confirmed in two of the four guano samples using Sanger sequencing and PCR for a 234 bp subfragment of the metagenomic contig (KM204383).

### Hepeviridae

**Fig. 5.**
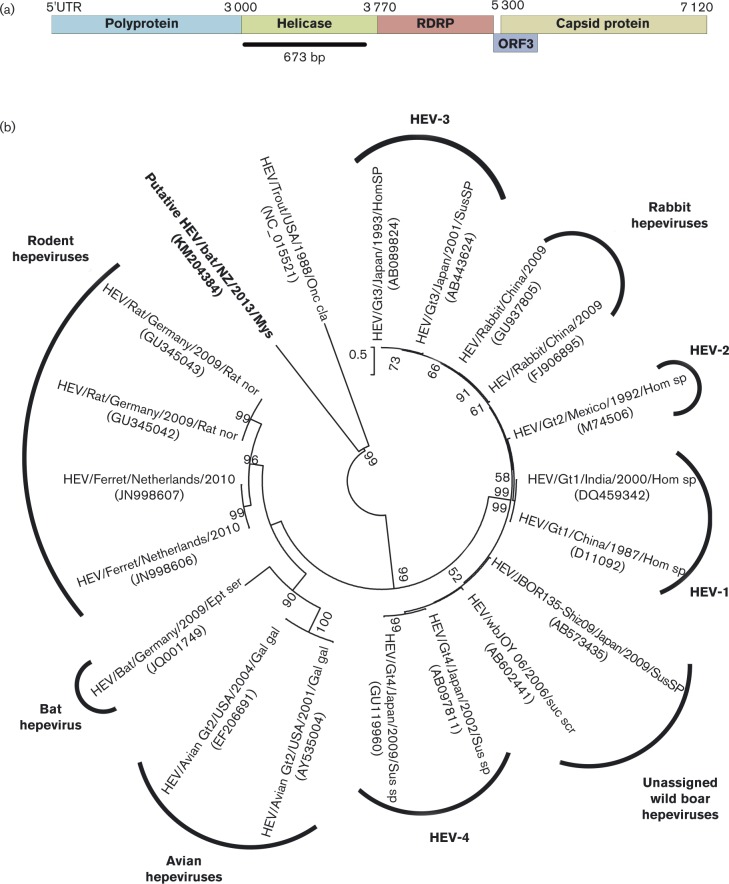
Identification of a putative hepevirus sequence. (a) Schematic representation of the location of contigs matching the hepevirus genome, shown as the lower black bar. (b) Phylogenetic tree showing the relationship of other hepeviruses with that of the New Zealand bat hepevirus helicase sequence of 224 aa (GenBank accession no. KM204384) obtained directly from the metagenomic data. The tree was compiled using the maximum-likelihood method and the JTT model with 1000 bootstrap replicates. Bootstrap values < 50 % are not shown. Bar, number of amino acid substitutions per site.

In total, 156 reads from the RNA metagenome were assembled into eight contigs that showed similarity to the helicase gene of hepeviruses. Phylogenetic analysis of the longest contig of 673 bp (GenBank accession no. KM204384) determined that the closest relative was the cut-throat trout virus (Fig. 5), a proposed new genus within the family *Hepeviridae* (Batts *et al.*, 2011). The New Zealand hepevirus amino acid sequence shared 30.3 % identity with its closest relative, the cut-throat trout virus (GenBank accession no. NC_015521). A 211 bp subfragment of this contig was confirmed in the RNA of all four original guano samples by using PCR and Sanger sequencing (GenBank accession no. KM204385).

### Other viruses

RAPSearch2 matches were also obtained for poxvirus, parvovirus, adenovirus and picornavirus (Table S1, available in the online Supplementary Material). No conserved genetic elements were identified for adenovirus or poxvirus. Of the 139 sequences assigned to poxvirus, the majority of the contigs and singleton reads were most closely related to molluscum contagiosum virus (MCV), a pathogen in humans. The MCV-like contigs and singleton reads showed between 55.6 and 75.4 % identity with another bat MCV-like sequence (GenBank accession no. NC_001731) at the protein level.

Members of the family *Parvoviridae* are known to infect a diverse range of hosts, including insects, and can be present as endogenous elements in the host genome. For this reason, parvoviruses were not subject to further investigation (Liu *et al.*, 2011).

Six contigs matching members of the family *Picornaviridae* were identified, but only one contig contained a viable ORF. This contig only encoded 116 aa (349 bp), matching the 2C gene. It was therefore excluded from further investigation given that it encoded fewer than 150 aa, the threshold set as a requirement for phylogenetic analysis and PCR confirmation (see Methods).

## Discussion

This study provides the first report of the virus diversity in the New Zealand lesser short-tailed bat, *M. tuberculata*. Despite extensive biogeographical isolation from other terrestrial mammals for more than 16 million years (Hand *et al.*, 2013), the viral sequences observed in the metagenomic data were generally consistent with those observed in other metagenomic studies of bat guano. Sequences with similarity to known vertebrate viruses were identified, including poxvirus, parvovirus, papillomavirus, calicivirus, hepevirus, polyomavirus and picornavirus (and also coronavirus from a previous study; Hall *et al.*, 2014a). It is possible that these viral sequences could have arisen as a consequence of dietary origin or subsequent to the deposition of guano by the bats, and this must be taken into consideration when interpreting these results. In addition, the polyomavirus and papillomavirus sequences were found only in the DNA virus metagenomes, so there is the possibility that these sequences were detected as endogenous/integrated elements from the host genome.

Metagenomic studies in bats from other countries have identified adenovirus (Baker *et al.*, 2013; Drexler *et al.*, 2011; He *et al.*, 2013; Li *et al.*, 2010a, b; Sonntag *et al.*, 2009; Wu *et al.*, 2012), papillomavirus (Ge *et al.*, 2012; Tse *et al.*, 2012b; Wu *et al.*, 2012), parvovirus (Ge *et al.*, 2012; Li *et al.*, 2010a) and a calicivirus (sapovirus, reported by Tse *et al.*, 2012a). It is difficult to make specific or direct comparisons with the present study due to significant methodological variation among the studies including: sample type (i.e. fresh guano, urine, roost guano), virus enrichment procedures (i.e. nuclease-treatment, centrifugation, filtration), and amplification methods and/or high-throughput sequencing platforms (Daly *et al.*, 2011; Hall *et al.*, 2014b). Perhaps the most comparable study is that of Baker *et al.* (2013), who used a metagenomic approach to examine virus diversity in African straw-coloured fruit bats (*Eidolon helvum*) (Baker *et al.*, 2013). Similar to the present study, *E. helvum* samples (lung tissue, urine and throat swabs) contained novel adenovirus, polyomavirus, papillomavirus and MCV-like sequences (Baker *et al.*, 2013). The mean pairwise identity of all MCV-like amino acid sequences from *M. tuberculata* compared with *E. helvum* was 65.5 %, suggesting that the New Zealand bats may host a different species of an MCV-like virus. Full-genome sequencing, or at least sequencing of the conserved major capsid protein, will be necessary before any provisional statement on species assignment of the MCV-like virus from *M. tuberculata* can be made.

Despite the large number of virus taxa indicated in the metagenomic data in the present study, a conservative approach was taken where: (i) only conserved genetic elements were considered, such as the capsid protein or helicase, and (ii) independent confirmation of the metagenomic data was required by specific PCR assay and Sanger sequencing of the amplicon. Given the proliferation of virus metagenomic studies, and high-profile instances of erroneous reporting of novel viruses (Naccache *et al.*, 2014), the use of conserved genes with an independent PCR assay and Sanger sequencing as well as appropriate controls should be considered minimum requirements before reporting the discovery of new virus sequences (Rosseel *et al.*, 2014). The most distinctive virus sequences found in this study were similar to those of caliciviruses and hepeviruses. This study is, to the best of our knowledge, only the second report of caliciviruses in bats (the first being the detection of a novel sapovirus; Tse *et al.*, 2012a).

A major review of the family *Caliciviridae* removed the hepatitis E viruses from this family, placing them as the sole genus within the family *Hepeviridae* (Berke *et al.*, 1997; Berke & Matson, 2000; Green *et al.*, 2000). The discovery of a hepatitis E virus in cut-throat trout (Batts *et al.*, 2011) has raised the possibility of an aquatic lineage of hepatitis E viruses, with the possible suggestion that this is a new genus (Batts *et al.*, 2011; Smith *et al.*, 2013). The putative hepevirus reported for *M. tuberculata* formed an alternate clade alongside the cut-throat trout hepevirus, separate from all other hepeviruses (Fig. 5b). The current classification for hepeviruses includes separate clades for rodent, bat, human and avian viruses, and it is postulated that co-divergence with the host has led to this (Drexler *et al.*, 2012). Therefore, the divergent hepevirus in *M. tuberculata* could be accounted for by co-speciation of all other hepeviruses subsequent to the isolation of *M. tuberculata* on the Zealandia subcontinent over 16 million years ago.

*M. tuberculata* is listed as a vulnerable species on the IUCN Red List (http://www.iucnredlist.org/) and has an important status for the indigenous Ma¯ori population of New Zealand. It is not possible to examine any of the viruses within an experimental colony. For any of the viruses identified, culture could be attempted either in specific bat cell lines or in other permissive cell lines such as Vero. Furthermore, complete genome sequencing will allow a greater degree of certainty in taxonomic assignment and will provide additional insights into the evolutionary history of viruses in *M. tuberculata*. Given the more recent arrival of the related native bat species *C. tuberculatus* (the long-tailed bat) approximately 1 million years ago, it will be interesting to explore the viral metagenome within this species to search for evidence of these viruses. An expansion in the survey of viruses in *M. tuberculata* is also warranted, as the present study relied upon just four guano samples collected from one island at one time point. Indeed, given that a broad survey of viruses in both rodent and chiropteran species in New Zealand has never been conducted (there are three introduced rat species in New Zealand: *Rattus rattus*, *Rattus exulans* and *Rattus norvegicus*), and that similar surveys in other countries have found an abundance of virus diversity (Li *et al.*, 2010a; Phan *et al.*, 2011; Tse *et al.*, 2012a), there is clearly a need to look more closely. This recommendation holds for any similarly remote or isolated area of the world. Whilst it is clear that emerging ‘hotspots’ of infectious disease require comprehensive surveys for virus diversity (Anthony *et al.*, 2013; Jones *et al.*, 2008), there may be additional value in examining low-diversity, remote ecosystems, as virus data could inform both the origins of virus taxa and provide a simplified model for the study of evolutionary processes such as co-divergence and cross-species transmission.

## Methods

### Sample collection

The collection protocols have been described previously (Hall *et al.*, 2014a). For this study, four bat guano samples were collected from each of four separate bat roosts on Codfish Island (Whenua hou) (Fig. S1, 46° 47′ S 167° 38′ E). Samples were kept at 4 °C and nucleic acids were extracted within 48 h. *M. tuberculata* is found only in New Zealand and is listed as ‘nationally endangered’. Relatively small populations of this bat are present only in remote areas of unmodified old forest. The bats are highly secretive and difficult to locate, as they frequently change roost sites and also roost in trees upwards of 15 m above the forest floor. Codfish Island is an active conservation study site where movements of this bat species are well known, and thus allowed the collection of fresh guano. Access to Codfish Island is restricted to scientists and conservation staff.

### Sample preparation and metagenomic sequencing

Bat guano was resuspended in 2 ml of sterile PBS, followed by centrifugation at 6000 ***g*** for 5 min. Nucleic acid was extracted from 400 μl of supernatant using an iPrep Purelink Virus kit (Life Technologies), with elution into 50 μl of RT-PCR molecular-grade water (Ambion). DNA and RNA were co-purified during extraction.

Given resource constraints, only two of the guano samples were selected for metagenomic sequencing, and the remaining two samples were used only for confirmatory PCR assays. For the purpose of finding RNA viruses, DNA was removed using DNase treatment by Ambion DNA-free (Life Technologies), and 8 μl of DNA-free RNA was incorporated into first-strand cDNA synthesis primed by random hexamers (Life Technologies), including an RNase H digestion. This step was not required for the complementary approach that sought to find DNA viruses. The cDNA and DNA from previous steps were then amplified by multiple displacement amplification using a Whole Transcriptome Amplification kit (Qiagen) to ensure that >1 μg of DNA was produced. This is the minimum amount required for library preparation using an Illumina TruSeq DNA Library Preparation kit. DNA libraries were prepared and sequenced on an Illumina MiSeq2000 instrument producing 250 bp paired-end reads (New Zealand Genomics Limited). Samples were indexed using barcodes so that each metagenome could be linked specifically to the original DNA and RNA.

### Bioinformatics workflow

FastQC (Andrews, 2010) was used to check sequence data quality, with reads trimmed using the FASTX-Toolkit with a base pair quality score threshold of 28 (http://hannonlab.cshl.edu/fastx_toolkit/index.html). Trimmed reads were then compared with the NCBI non-redundant protein sequence database (downloaded in April 2013) using RAPSsearch2 (Ye *et al.*, 2011; Zhao *et al.*, 2012), an alternative to blastx (Altschul *et al.*, 1990). Taxonomic assignment of the RAPSearch2 output was performed using megan 5.2 (Huson *et al.*, 2007; Huson & Mitra, 2012) with a threshold for assignment of bit scores >50 and read complexity >0.44. Paired-end read data of interest was then extracted and assembled into contigs in Geneious 6.1.7 (Biomatters; http://www.geneious.com).

### Confirmatory PCR

For conserved genes of interest from the metagenomic data that would allow further taxonomic assignment and phylogenetic analysis (i.e. vertebrate virus capsid, helicase or RNA-dependent RNA-polymerase gene regions), specific primers were designed using the Primer3 plug-in for Geneious 6.1.7. A 25 μl reaction volume of Invitrogen Platinum SuperMix (Life Technologies) was used as per the manufacturer's instructions, including 5 μl of DNA or cDNA. Primer sequences are detailed in Table S2. PCR products were visualized by agarose gel electrophoresis, followed by purification using USB ExoSAP-IT (Affymetrix) and Sanger sequencing on a capillary sequencer (model 3100 Avant; Applied Biosystems).

### Phylogenetic analysis

The nucleotide sequences produced by the confirmatory PCR assays were translated into protein sequences and compared with reference virus genomes downloaded from GenBank. Only sequences with an ORF of >150 aa were considered for phylogenetic analysis. Sequences were aligned in mega6 (6.06) using clustal w with a gap opening penalty of 10 and gap extension penalty of 0.2 (Tamura *et al.*, 2011). Maximum-likelihood phylogenetic trees were reconstructed in mega6 using all available amino acid sites with 1000 bootstrap replicates and the nearest-neighbour-interchange method.

## Supplementary Data

487 Supplementary Data
