# Supplementary material for: Discovery of novel virus sequences in an isolated and threatened bat species, the New Zealand lesser short-tailed bat (Mystacina tuberculata)
Source: J Gen Virol. 2015 Aug;96(Pt 8):2442–52. doi: 10.1099/vir.0.000158 (PMC4681071; doi:10.1099/vir.0.000158)
Supplement: Supplementary file 1 — Supplementary Data [file vir-96-08-2442-s001.pdf]

In: *Discovery of novel virus sequences in an isolated and threatened bat species, the New Zealand lesser short-tailed bat (Mystacina tuberculata)* . Wang J, Moore NE, Murray ZL, McInnes K, White DJ, Tompkins DM, Hall RJ*. Journal of General Virology. (*Corresponding author : Richard J. Hall, Institute of Environmental Science & Research, richard.hall@esr.cri.nz)

Table S1. RAPSearch2 similarity statistics for reads matching other vertebrate virus in New Zealand lesser short-tailed bats.

Virus	Read _ID	Contigs / Read Length (bp)	Score	e-value	% query identity	% query coverage	Best hit	Gene
Adenovirus	M00933:46:000000000-A2BMW:1:1105:9416:7977/1	250	85.9	2.E-18	49	94	AP_000407	ORF13[Fowl adenovirus A]
	M00933:46:000000000-A2BMW:1:1105:9416:7977/2	250	99	4.E-23	51	99	AP_000407	ORF13[Fowl adenovirus A]
	M00933:46:000000000-A2BMW:1:1110:9837:12704/2	250	76.3	6.E-15	57	69	AP_000407	ORF13[Fowl adenovirus A]
Molluscum contagiosum virus	Contigs 1	583	296	5.E-46	90	82	AAB57956	similar to variola H8R and vaccinia G8R [Molluscum contagiosum virus subtype 1]
	Contigs 2	809	431	2.E-51	76	95	AAB57958	similar to variola M1R and vaccinia L1R [Molluscum contagiosum virus subtype 1]
	Contigs 3	575	262	8.E-40	79	76	NP_044051	MC100R [Molluscum contagiosum virus subtype 1]
	Contigs 4	448	106	2.E-25	53	68	NP_044073	MC122L [Molluscum contagiosum virus subtype 1]
	Contigs 7	611	354	6.E-72	77	99	AAB57999	similar to variola A25R and vaccinia A24R [Molluscum contagiosum virus subtype 1]
	Contigs 8	476	122	8.E-29	86	41	NP_044080	MC129R [Molluscum contagiosum virus subtype 1]
	Contigs 9	382	89.4	2.E-19	93	33	AAB57949	similar to variola and vaccinia E4L [Molluscum contagiosum virus subtype 1]
	Contigs 14	281	93.2	7.E-20	56	99	NP_044051	MC100R [Molluscum contagiosum virus subtype 1]
	Contigs 15	445	156	9.E-22	53	99	BAA25417	70-kDa protein [Molluscum contagiosum virus]
	Contigs 16	411	177	3.E-35	54	99	NP_044041	MC090R [Molluscum contagiosum virus subtype 1]
	Contigs 17	401	249	4.E-75	86	99	NP_044045	MC094R [Molluscum contagiosum virus subtype 1]
	Contigs 18	392	193	8.E-35	54	100	NP_044038	MC087R [Molluscum contagiosum virus subtype 1]
	Contigs 19	389	99.8	3.E-22	62	59	NP_044079	MC128R [Molluscum contagiosum virus subtype 1]
	Contigs 20	386	246	5.E-75	94	99	NP_044046	MC095R [Molluscum contagiosum virus subtype 1]
	Contigs 21	385	234	1.E-70	80	99	NP_044051	MC100R [Molluscum contagiosum virus subtype 1]
	Contigs 22	382	111	6.E-27	66	62	NP_044023	MC072L [Molluscum contagiosum virus subtype 1]
	Contigs 23	376	149	1.E-40	56	99	AAB57954	similar to variola and vaccinia E9L [Molluscum contagiosum virus subtype 1]
	Contigs 24	375	141	5.E-37	64	81	NP_043982	MC031L [Molluscum contagiosum virus subtype 1]
	Contigs 25	363	242	3.E-72	93	100	NP_044041	MC090R [Molluscum contagiosum virus subtype 1]
	Contigs 26	357	180	9.E-37	87	95	NP_044036	MC085L [Molluscum contagiosum virus subtype 1]
	Contigs 27	349	184	3.E-51	85	90	NP_044030	MC079R [Molluscum contagiosum virus subtype 1]
	Contigs 28	346	100	4.E-25	73	54	NP_044012	MC061R [Molluscum contagiosum virus subtype 1]
	Contigs 30	339	208	3.E-61	85	100	NP_044051	MC100R [Molluscum contagiosum virus subtype 1]
	Contigs 34	305	112	7.E-26	51	95	NP_043986	MC035R [Molluscum contagiosum virus subtype 1]
	Contigs 35	294	90.5	7.E-19	50	77	AAB58003	similar to variola A30L and vaccinia A27L [Molluscum contagiosum virus subtype 1]
Parvovirus	Contigs	277	55	1.E-06	41	79	AHA86835	Nonstructural protein [Seal parvovirus]
	M00933:46:000000000-A2BMW:1:2103:21012:5912/2	250	55.1	1.E-06	59	46	AAB82734	capsid protein[chipmunk parvovirus]
	M00933:46:000000000-A2BMW:1:2104:22426:10197/2	250	84.7	5.E-17	56	76	AGC92300	VP1[Human bocavirus]
Picornavirus	Contigs 1	477	113	3.E-13	43	74	AGU62957	polyprotein[Ferret parechovirus]
	Contigs 3	567	170	8.E-48	47	93	ADR79388	polyprotein[Picorna-like virus Eptesicus fuscus/P5/InLV/IT/USA/2009]
	Contigs 4	571	114	8.E-27	53	75	AIM55450	NS, partial [Ancient Northwest Territories cripavirus]
	Contigs 8	349	61.6	2.E-08	42	74	ABO09966	polyprotein [Duck hepatitis virus 2 strain 90D]
	Contigs 9	331	65.9	3.E-10	53	48	AIM55450	NS, partial [Ancient Northwest Territories cripavirus]
	Contigs 11	294	52	2.E-05	45	47	AFR11852	polyprotein [Salivirus sewage Bangkok]

In: *Discovery of novel virus sequences in an isolated and threatened bat species, the New Zealand lesser short-tailed bat (Mystacina tuberculata).* Wang J, Moore NE, Murray ZL, McInnes K, White DJ, Tompkins DM, Hall RJ*. Journal of General Virology. (*Corresponding author : Richard J. Hall, Institute of Environmental Science & Research, richard.hall@esr.cri.nz)

Table S2. Primer pairs used for amplification of target viral genes.

Virus	Target gene	Forward primer name	Forward Primer Sequence	Reverse Primer Name	Reverse Primer Sequence	Product size (bp)
Papillomavirus	Major capsid protein (L1)	Papillomavirus_L1_423F	CCCTGGTGTTATCCCCAACC	Papillomavirus_L1_941R	AGCTGGGGGAGTATTGGACT	517 / 522
Polyomavirus	Viral protein 1	Polyvirus_VP1_375 F	GGCTGTTTGTTTCTGCTGCA	Polyvirus_VP1_561 R	TGCCCCTCCATACTAGGCAT	187
Calicivirus	Major capsid protein	Cal56F	GATCGTTCAACGTTGCCACC	Cal357R	GTCTGGGGCTGTGAACATGA	302
	Helicase-like protein	Cal36F	ACAGCAGCAGATGTTTCAGT	Cal269R	TCCACGTACTGGCAAAGATGAA	234
Hepevirus	Helicase protein	HEV6F	GTTTCGCTAACGCCCATACC	HEV215R	ATATGCCTTCCGCTACAGGC	211

In: *Discovery of a novel calicivirus, hepevirus and other vertebrate viruses in an isolated and threatened bat species, the New Zealand lesser short-tailed bat (Mystacina tuberculata).* Wang J, Moore NE, Murray ZL, McInnes K, White DJ, Tompkins DM, Hall RJ*. Journal of General Virology.
(*Corresponding author : Richard J. Hall, Institute of Environmental Science & Research, richard.hall@esr.cri.nz)

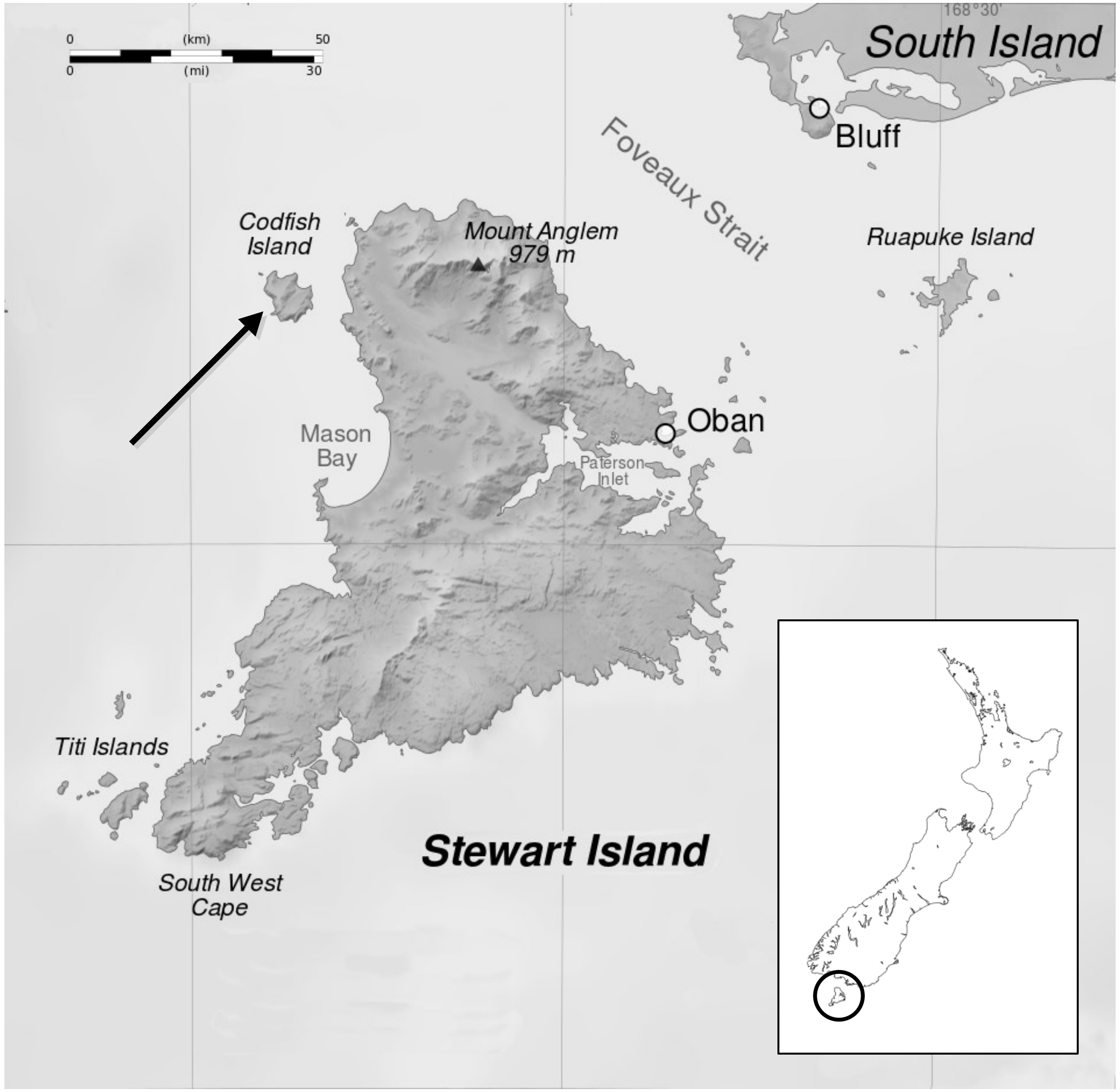

Fig. S1. Location of Whenua hou / Codfish Island. Map of New Zealand showing the location of Codfish Island / Whenua hou where bat guano samples were collected. *Mystacina tuberculata* is currently the sole terrestrial mammalian species (except for humans).
